# Clinical and radiological assessment of grape seed extract versus mineral trioxide aggregates in primary molar pulpotomy: A randomized controlled clinical trial

**DOI:** 10.1097/MD.0000000000044827

**Published:** 2025-09-26

**Authors:** Noura Mohammed Bakr, Gihan Adel Balbola, Soha Fuad Alqadi, Najla Dar-Odeh, Shadia Elsayed, Nuha Alghamdi, Nisreen Nabiel Hassan, Sara N. Hashem, Safaa Ramadan El Sayed

**Affiliations:** a Department of Oral and Dental Biology, Faculty of Dental Medicine for Girls, Al-Azhar University, Cairo, Egypt; b Department of Oral Pathology, Faculty of Dental Medicine for Girls, Al-Azhar University, Cairo, Egypt; c Department of Preventive Dental Sciences, College of Dentistry, Taibah University, Madinah, Saudi Arabia; d Department of Restorative Dentistry, College of Dentistry, Taibah University, Madinah, Saudi Arabia; e Department of Oral and Maxillofacial Surgery, Oral Medicine and Periodontology, School of Dentistry, The University of Jordan, Amman, Jordan; f Department of Restorative Dental Sciences, College of Dentistry, King Khalid University, Abha, Saudi Arabia; g Department of Pedodontics and Oral Health, Faculty of Dental Medicine for Girls, Al-Azhar University, Cairo, Egypt.

**Keywords:** dental material, Grape seed, herbal, MTA, primary teeth, pulp tissue, pulpotomy

## Abstract

**Background:**

This study aimed to assess the radiological and clinical outcomes of mineral trioxide aggregates (MTA) versus grape seed extract (GSE) when used as a dressing material in primary teeth undergoing pulpotomy.

**Methods:**

This was a prospective split-mouth randomized clinical study. A total of 32 primary teeth were included and split into 2 groups: MTA was used in group I, and GSE was used in group II. A clinical and radiographic follow-up was conducted for 6 months postoperatively. Periapical radiographs were taken for all treated teeth at 3- and 6-month follow-up visits. Radiographic criteria for clinical success included absence of internal resorption, pathologic external resorption, interradicular radiolucency, periapical radiolucency, and widening of the periodontal ligament space.

**Result:**

In the MTA group, clinical and radiographic success was 100% at 1 and 3 months, in comparison to 93.75% 6 months postoperatively. In the GSE group, clinical success was 100% 1 month, and 93.75% 3 and 6 months postoperatively, while radiographic success was 100% at 3 months, 87.5% 6 months postoperatively. There were no significant statistical differences in the overall clinical or radiographic outcomes between the study groups (*P* > .05).

**Conclusion:**

Both MTA and GSE are associated with successful clinical and radiographic outcomes of pulpotomy; however, MTA seems to be associated with reduced inflammatory reactions, rendering it more effective than GSE.

## 1. Introduction

Dentine formation during the growth of teeth depends in part on pulp and additionally in the development of reparative, sclerotic, and reactive dentin in the case of pathological stimulation after tooth eruption. Exposed pulp can recover through cell reorganization and dentin bridge formation if micro-leakage is effectively prevented, Direct pulp capping with various materials represents one of these preventive measures.^[1]^ Pulpotomy is a favorable type of treatment for asymptomatic pulp in carious teeth without root involvement, which prevents early loss of teeth. The choice of an efficient and biocompatible pulp capping material is as important as accurate identification of pulpitis for a successful pulpotomy procedure.^[2]^ Bactericidal material that can be used for pulp capping, should encourage pulp tissue healing, and, at the same time, should not impede naturally-occurring root resorption, and should be safe for cells and nearby structures.^[3]^

Mineral trioxide aggregates (MTA), have been so far a reliable pulp capping material which was reported to be associated with a thicker dentin bridge, and less adverse outcomes such as pulp hyperemia and inflammatory reaction. This material which is composed of calcium sulphate, bismuth oxide, tricalcium aluminate, tricalcium silicate, and dicalcium silicate, releases primary calcium ions that reacts with phosphates to grant MTA the required characteristics of biocompatibility and sealing capacity.^[1,4]^

Despite the reported biocompatibility profile of MTA, the quest for natural dentin biocompatible material has been the demand of several researchers who were looking for natural alternatives to be used for the pediatric population.^[5,6]^ Grape seed extract (GSE) is a potential candidate for pulp capping. It is a rich source of proanthocyanidins (PACs) and is composed of monomeric catechin and epicatechin, gallic acid and polymeric and oligomeric PACs.^[7,8]^

Based on the interaction of PACs with dentin collagen, GSE has previously shown its biological potential for promoting increased dentin hardness. This substance has anti-inflammatory and antioxidant properties. Collagen cross-linking has already been demonstrated to support collagen-based tissues. There is evidence that PACs raise the rate at which soluble collagen becomes insoluble collagen and increases collagen synthesis during development.^[9]^

It has been demonstrated in earlier studies that GSE performed well and improve the physical characteristics, lowers the levels of dentin degradation in healthy and damaged dentin. Furthermore, it has the potential to engage in interactions involving dentin collagen. By inhibiting proteases like intrinsic metalloproteinases, GSE may also contribute to reducing collagen degradation.^[10,11]^

Treatment using GSE has been reported to have no direct or indirect cytotoxic effects on cells of human pulp even at high concentrations for long periods. When in close proximity to the pulp cells, components of GSE may provide protection and bio-stimulatory effects.^[12]^ Therefore, this study was conducted to perform clinical, and radiological assessment of the biological response of dental pulp tissues when exposed to GSE and MTA capping materials in primary teeth pulpotomy.

## 2. Material and methods

### 2.1. Study design and inclusion criteria

This prospective split-mouth randomized clinical trial cohort study was conducted on the 32 extremely carious (first and/or second) primary molars that were indicated for pulpotomy. The Human Research Ethics Committee of the Faculty of Dental Medicine at Al Azhar University for Girls approved this study (REC-PD-23-01). All Participants’ Parents or guardians signed a written formal consent form before surgery.

**Figure 1.**
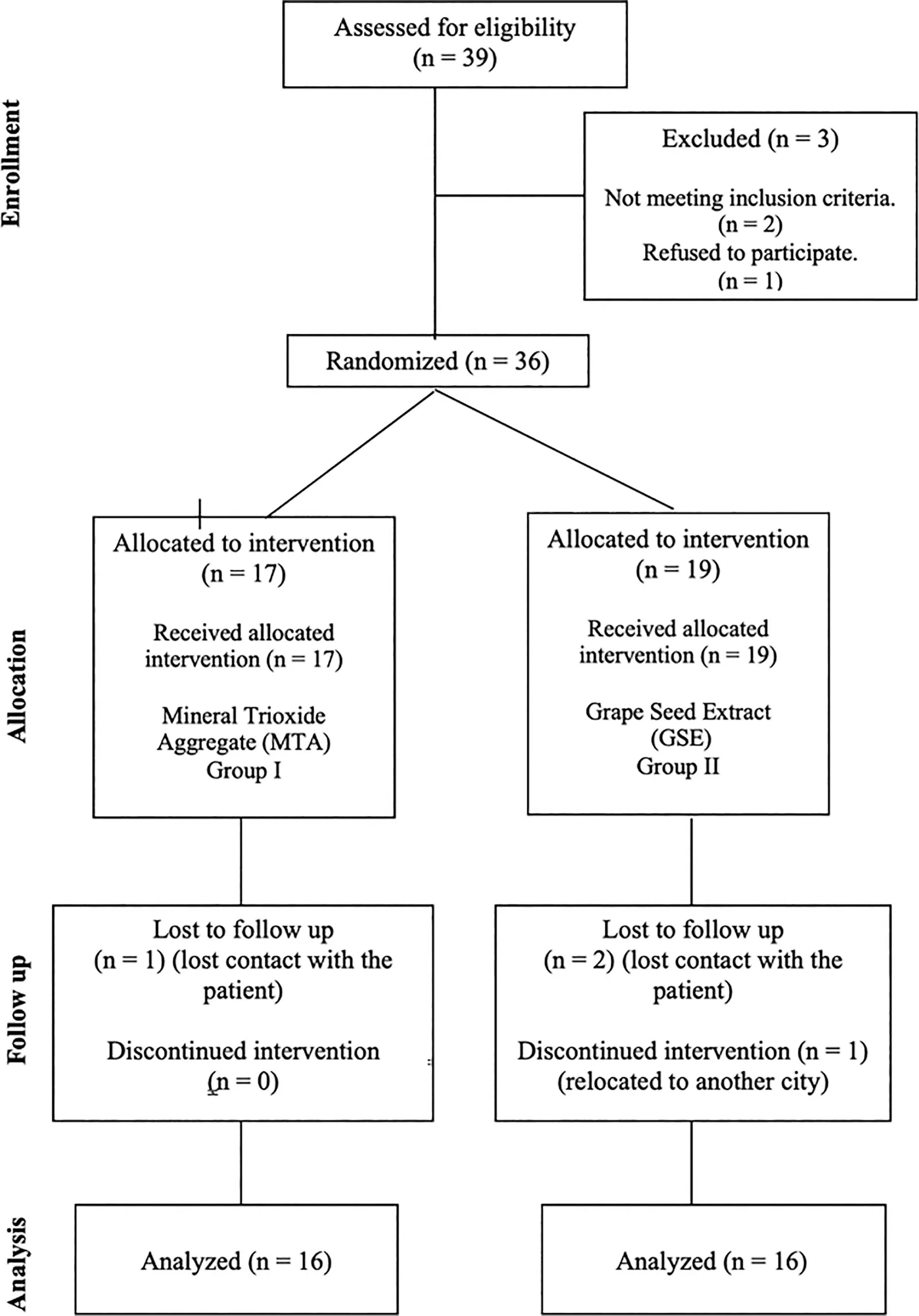
CONSORT diagram illustrating the flow of participants through each stage of a randomized trial. CONSORT = consolidated standards of reporting trials, GSE = grape seed extract, MTA = mineral trioxide aggregates.

The study protocol record was registered on ClinicalTrials.gov with the identifier (NCT06694064). The current research was conducted in accordance with the Helsinki Declaration Principles laid down in 1975, and reported according to consolidated standards of reporting trials guidelines, as shown in the flow diagram (Fig. 1). Inclusion criteria were as follows: at least 2 corresponding bilateral carious primary molars that are indicated for pulpotomy, cooperative and healthy pediatric patients, absence of clinical indicators of pulp degeneration, such as the history of spontaneous tenderness, pain on percussion, edema, pathological movement, or sinus tract. Pulp exposure was attributed to mechanical trauma rather than caries. Further, hemorrhage from the amputated pulp stumps should be easily controlled using sterile, moist cotton pellets in <5 minutes.

### 2.2. Exclusion criteria

Exclusion criteria included medically compromised children; a history of allergy to latex, local anesthetics, or the components of the pulp dressing agents; teeth with caries in close proximity to the pulp; clinically and radiographically healthy periodontal tissue; at least 2-thirds of the root is still remaining; no signs of periapical or furcal radiolucency, widening of the periodontal ligament, or pathological root resorption (internal or external).

### 2.3. Sample collection

For the purpose of determining sample size, the G power statistical power Analysis program (version 3.1.9.7) was used.^[13]^ A 2-sided hypothesis test with a total sample size of 32 (16 in each group) was found to be capable of detecting an effect size of 0.5 with an actual power (1-error) of 0.8 (80%) and a significance level (error) of 0.05 (5%).^[14]^

### 2.4. Statistical method

The XCP extension cone paralleling technique was employed to take a preoperative periapical radiograph when using a saliva ejector, rubber dam and achieve deep local anesthesia. One investigator (N.B.) operated on teeth including cavity preparation, placement of pulp capping material and glass ionomer restoration. Both groups received a conventional pulpotomy intervention in 1 visit. A large, slow-speed round bur was used to remove dentin, soft debris, and caries. The pulp chamber was then de-roofed and the overhanging dentin was removed using a diamond round bur mounted in a water-cooled high-speed hand piece. Using a sharp, large, sterile spoon excavator, the inflamed coronal pulp tissues were amputated up until the entrance of the roots, then gently debrided with ordinary saline irrigation, and finally dried with sterile cotton pellets.

The study molars were randomized into one of the 2 treatment groups: group I (MTA): the pulp was treated by MTA (MTA, CW9, Matreva, white MTA, Egypt). Group II (GSE): Grape seed powder purchased from Herb store (USA.com, Walnut). According to the data which was provided by the manufacturer, it consisted of 95% PAC. Catechins were the main monomers available in PAC. In this study, 6.5% (w/v) solution in phosphate buffer (0.025 M KH_2_PO_4_, pH 7.4) was used. Grape seed solution was prepared from the same lot of the extract. We mixed distilled water with GSE powder forming dark red thick mix of grape seeds filling. All pulpotomized molars’ prepared cavities were sealed with a layer of dressing materials and then filled with glass-ionomer restoration (Ketac Molar, 3M ESPE).

Patients were instructed to contact the investigator if any adverse signs or symptoms occurred between follow up visits. The children were recalled for clinical examination after 3 and 6 months, to check for any signs and symptoms in the treated teeth. One operator who was blinded to materials used in the test groups (Gihan Balbola) performed the clinical and radiographic assessment. Teeth showing the following criteria were considered as success; no symptoms of pain, no swelling of pulpal origin, no fistula, no tenderness to percussion, no pathological mobility. The clinical assessment was done at each follow up visit; data were recorded in a patient evaluation form. Periapical radiographs also were taken for all treated teeth at 3 and 6 months follow up visits only using the same standardized technique described before for preoperative radiographs. The following radiographic criteria were considered an indication for successful treatment outcomes: no internal resorption, no pathologic external resorption, no interradicular radiolucency, no periapical radiolucency, no widening of periodontal ligament space.

## 3. Results

The study included 16 children: 7 males and 9 females, with age range 4 to 8 years and mean (5.59 ± 1.18). A total of 16 teeth per each group were included in this study. In the MTA group clinical success and overall success was 100% at 1 and 3 months, in comparison to 93.75% after 6 months. There was no discernible difference by time (*P* = .360). Radiographic success was 100% at 3 months, in comparison to 93.75% after 6 months, with no significant difference by time (*P* = .310; Table 1).

**Table 1:** Clinical and radiographic success rates of treatment groups and statistical significance of differences between groups.

	Clinical success			Radiographic success			Overall success		
	Group (I) MTA	Group (II) GSE	*P* value (intergroup)	Group (I) MTA	Group (II) GSE	*P*-value (intergroup)	Group (I) MTA	Group (II) GSE	*P*-value (intergroup)
1 mo	16 (100%)	16 (100%)	–	–	–	–	16 (100%)	16 (100%)	–
3 mo	16 (100%)	15 (93.75%)	0.310	16 (100%)	16 (100%)	–	16 (100%)	15 (93.75%)	.310
6 mo	15 (93.75%)	15 (93.75%)	–	15 (93.75%)	14 (87.5%)	.540	15 (93.75%)	14 (87.5%)	.540
*P* value (intragroup)	.360	.590		.310	.140		.360	.340	

Significance level *P* ≤ .05, ns = non-significant.

GSE = grape seed extract, MTA = mineral trioxide aggregates.

**Figure 2.**
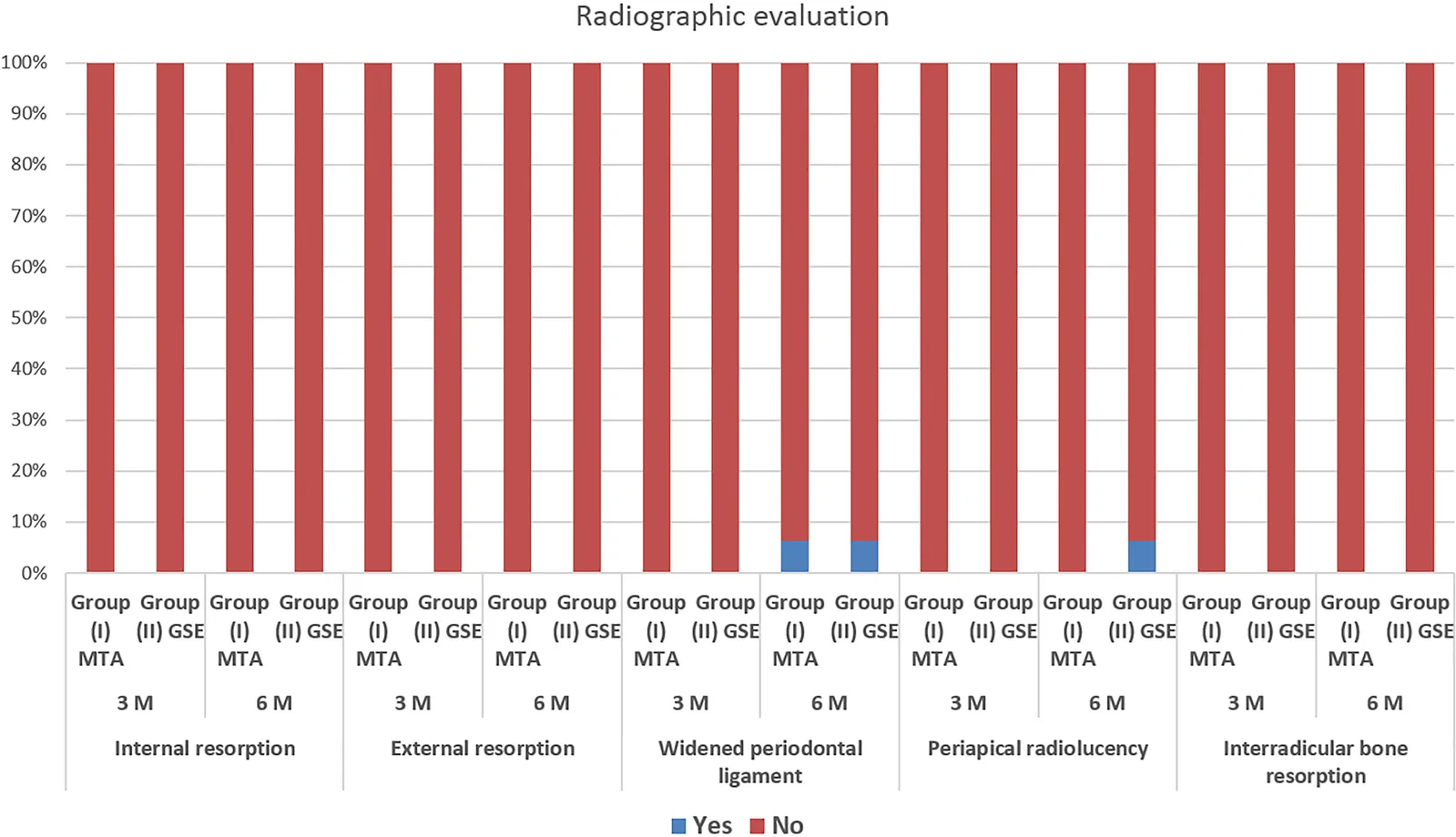
Bar chart illustrating results of radiographic evaluation at 3 and 6 mo postoperatively. GSE = grape seed extract, MTA = mineral trioxide aggregates.

In GSE group clinical success was 100% at 1 month, in comparison to 93.75% after 3 and 6 months, and there was no discernible difference by time (*P* = .590). Radiographic achievement was 100% at 3 months, in comparison to 87.5% after 6 months, with no significant difference by time (*P* = .140; Fig. 2). Overall success was 100% at 1 month, in comparison to 93.75% after 3 months and 87.5% at 6 months, with no discernible difference between groups (*P* = .340; Table 1).

## 4. Discussion

Pulpotomy is the most frequently employed vital pulp therapy technique in carious primary molars.^[15]^ In a substantial proportion of countries with low resource settings, children often lose primary molars at an early age, which compromises the proper functions of the oro-facial complex, including esthetics, mastication, and speech. Therefore, it is important for clinicians to use materials that are ideally bactericidal, safe, nontoxic to the pulp and nearby structures, and that encourage any remaining radicular pulp healing without interfering with physiological root resorption.^[16]^

This study utilized clinical and radiographic assessments for the treatment outcomes after using MTA or GSE. Clinical assessment showed a comparable and high success rate of pulpotomy in both groups in more than 90% of the study sample. However, despite the high success rate, radiographic assessment after 6 months showed a lower success rate of the GSE group than the MTA group. We could not follow the teeth for a longer duration, however, other studies estimated that the success rates of MTA at 24 months ranged from 75% to 100%.^[16]^ According to a previous study by Beltagy and Yasser, at 9 months radiographic success was observed in (80%) in manufactured material group, in comparison to (100%) in natural product group.^[14]^

Studies reported that the high alkalinity of MTA could be associated with mild pulp inflammation, however, it is reversible.^[6,17–19]^ MTA’s bioactivity induces remineralization, formation of dentin bridge, and produces transforming growth factor-β1.^[6,20]^ Osteocalcin and dentin sialoprotein, as well as increased secretion of vascular endothelial growth factor, were upregulated when MTA was in direct contact with dental pulp cells, according to Paranjpe et al^[21]^

Additionally, Alzoubi et al found that MTA bridge formation was more common than with portland cement.^[22]^ Al-Dlaigan reported similar findings, stating that in primary teeth pulpotomy, MTA performed better than formocresol, ferric sulphate, and calcium hydroxide in terms of treatment outcomes.^[23–25]^ The mechanism by which MTA induces dentin bridges is yet to be elucidated. This material has excellent biocompatibility and sealing capabilities.^[26–28]^ Furthermore, it has a high compressive strength and minimal solubility.^[29]^ MTA may work similarly to calcium hydroxide, according to some authors. As MTA hardens, it contains calcium oxide instead of calcium hydroxide, which can then react with tissue fluids to produce calcium hydroxide.^[24,30]^ As shown by Koh, MTA enhances the cytokine production of human osteoblasts.^[31]^ Mitchell postulated that MTA’s dentinogenic effect resulted from its excellent sealing ability and biocompatibility.^[27]^

On the other hand, fractions of natural extracts are hydrophilic, particularly those containing polyphenols, which have high-molecular-weight (PACs). PAC from GSE was shown to decrease the activity of amylase and glycosyltransferase F-ATPase, and stop the breakdown of organic materials in dentin matrix. Therefore, we used GSE in this study.^[32]^ Via the interaction of PA and collagen, the organic dentin of the root may be affected by GSE, stabilizing the matrix of exposed collagen. Interactions between PA and proteins are thought to be either hydrophobic, ionic, covalent, or hydrogen bonds. Demineralized dentin’s ultimate tensile strength was found to be significantly increased after GSE treatment, according to Bedran-Russo et al which suggests that cross-links in the collagen of the dentin may be caused by GSE.^[33]^ Pulp inflammation may either proceed to necrosis or to repair and healing if the equilibrium between damage and regeneration is not sustained. Control of oxidative stress and inflammation is warranted for enhancing stem cell proliferation, differentiation, and viability while preventing cell senescence. Grape seed PAs were shown in previous studies to enhance osteoblast differentiation and prevent bone loss by down regulating osteoblast apoptosis and osteoclast formation.^[34]^

Due to the small number of clinical trials that used GSE as a pulpotomy agent, methodology, variation in case selection criteria, the concentration of the extract materials which may affect the outcome, comparison with prior studies may be challenging. Even though our results are extremely encouraging, more studies for clinical and histological evaluation, along with longer follow-up evaluations, are needed to prove the safe clinical indication of GSE as an alternative herbal medicines.

## 5. Conclusion

Both MTA and GSE showed successful pulpotomy results on the clinical, radiographic, and histological aspects; nevertheless, MTA was still the most effective material for pulpotomies of primary teeth with reduced inflammatory reactions.

## Author contributions

**Conceptualization:** Noura Mohammed Bakr, Shadia Elsayed.

**Data curation:** Noura Mohammed Bakr, Gihan Adel Balbola, Soha Fuad Alqadi, Najla Dar-Odeh, Shadia Elsayed, Nuha Alghamdi, Safaa Ramadan El sayed.

**Formal analysis:** Gihan Adel Balbola, Soha Fuad Alqadi, Nisreen Nabiel Hassan, Sara N. Hashem.

**Funding acquisition:** Gihan Adel Balbola, Sara N. Hashem.

**Investigation:** Najla Dar-Odeh, Nuha Alghamdi, Nisreen Nabiel Hassan, Safaa Ramadan El sayed.

**Methodology:** Soha Fuad Alqadi, Najla Dar-Odeh, Shadia Elsayed, Nuha Alghamdi.

**Project administration:** Noura Mohammed Bakr, Safaa Ramadan El sayed.

**Resources:** Najla Dar-Odeh, Sara N. Hashem.

**Software:** Noura Mohammed Bakr, Soha Fuad Alqadi.

**Supervision:** Najla Dar-Odeh, Sara N. Hashem.

**Validation:** Gihan Adel Balbola, Shadia Elsayed, Nisreen Nabiel Hassan.

**Visualization:** Shadia Elsayed, Nisreen Nabiel Hassan, Sara N. Hashem, Safaa Ramadan El sayed.

**Writing** – **original draft:** Noura Mohammed Bakr, Soha Fuad Alqadi, Nisreen Nabiel Hassan, Safaa Ramadan El sayed.

**Writing** – **review & editing:** Noura Mohammed Bakr, Gihan Adel Balbola, Soha Fuad Alqadi, Najla Dar-Odeh.
